# An exploration of the editing cut as an articulator in film through frequency domain analysis of spectator EEGs

**DOI:** 10.3389/fnins.2025.1489800

**Published:** 2025-07-03

**Authors:** Javier Sanz-Aznar

**Affiliations:** Section of Communication, Department of Hispanic Studies, Literary Theory and Communication, University of Barcelona, Barcelona, Spain

**Keywords:** film, editing, ERD/ERS, EEG, cut, cinema, neurocinematics, cinematographic language

## Abstract

**Introduction:**

This study explores if the cinematographic cut can be considered an articulation axis between different units, which are adjacent shots. The theoretical premise of the research was that if the shot change functions as a point of articulation that produces a connection between different units, two conditions must be met: first, all types of editing cuts should elicit common neural patterns; and second, these neural patterns triggered to make sense of the shot change should exhibit variations depending on the specific type of cut.

**Methods:**

To achieve this objective, building on the theoretical foundations of cinematographic language and integrating methodologies from cognitive neuroscience, the study analyzed neural responses triggered by continuity editing cuts through electroencephalography recordings from 21 participants.

**Results:**

To determine it, the frequency domain of spectators’ neural recordings were analyzed for common event-related desynchronization/synchronization patterns. The analysis revealed neural responses patterns in theta synchronization and delta desynchronization, which are associated with memory encoding, narrative segmentation, and meaning construction.

**Discussion:**

Moreover, the results suggests that shot changes are cognitively processed as relational events, not merely as new perceptual inputs. These findings support the hypothesis that the shot change by cut is neurally processed as an articulatory mechanism within film structure.

## 1 Introduction

The analysis of the shot change by cut is approached from a cognitive ecological perspective (Anderson, 1996) has gained increasing attention in the last years (Ben-Yakov and Henson, 2018; Bálint et al., 2020; Loschky et al., 2020). This approach emphasizes how the cognitive gap of the shot change is naturally —and even imperceptibly— assimilated narratively by the spectators. This approach to studying film editing, which includes experimental techniques that allow for the validation or refutation of specific proposals, predominantly addresses two fundamental aspects. The first consists of studying how cinematographic technique allows the cut to go unnoticed by the viewer’s consciousness (Smith and Henderson, 2008), and the second focuses on how the fragmentation into different shots is understood by the viewer, taking the set of shots as a unit through narrative integration and the comprehension of fragmented events as a single continuous entity (Loschky et al., 2020).

The main study about the feel of continuity in the viewer despite a shot change was developed by Tim Smith (Smith and Henderson, 2008; Smith, 2005; Smith, 2012; Smith, 2013), which gave rise to the Attentional Theory of Cinematic Continuity (AToCC). This proposal, developed in various publications by Smith, addresses how classical rules of film editing match with natural attentional cues such as conversational turns, motion continuity or gaze cues. The combination of these kinds of attentional cues around the cut matching minimal expectations creates the sensation of invisibility of the cut in the viewer, facilitating an uninterrupted virtuality in which to develop the film narrative. The AToCC helps to understand how the technical-narrative device of film editing enables the development of a continuous narrative despite its construction being inherently fragmented.

Regarding the assimilation of the film’s narrative content by the spectator despite the fragmentation by cuts, it is of particular interest the Scene Perception and Event Comprehension Theory (SPECT) applied to visual narratives (Loschky et al., 2020). Through the Event Indexing Model (Magliano et al., 2001) and the Event Comprehension Theory applied to visual narratives (Loschky et al., 2020), an explanation emerges about how viewers generate cognitive models while watching a movie, thus being able to assimilate the narrative continuously. These mental models linked to the narrative are continuously updated by identifying changes in the story entities and the event models. For the detection of these variations, a constant evaluation and comparison occur between schemas and processes (Bordwell, 1991; Tan, 2018) top-down (for the understanding of the scene) and bottom-up (defining the characteristics of the stimulus). For this constant flow to develop normally, it is necessary for a smooth progression of events to occur throughout the narrative, enhancing the absorption experience during the viewing of the film, especially when cuts appear that hinder bottom-up processes.

According to the proposal of the SPECT applied to visual narratives (Loschky et al., 2020), the shot change can break temporal and spatial coherence, requiring a reconstruction of the event model through bridging inferences, connecting information from previous events with the new ones. On the one hand, the perception of events in a visual narrative is segmented through cuts that mark clear boundaries between distinct events. This situation occurs, for example, in changes in space, time, or the characters’ objectives, creating event boundaries that clearly segment the narrative. Thus, the cut transition facilitates the shift between event models, as the cuts emphasize the discontinuities between events and allow the viewer to adjust their understanding of the story. On the other hand, editing has the capacity to organize the information associated with events in such a way that the viewer can construct a unique model through its segmentation into various shots. Therefore, the cut not only separates the events but also helps to structure the perception of the action and the narrative, allowing the spectator to integrate the new aspects while maintaining the coherence of the narration.

Although both AToCC and SPECT offer compelling models for understanding the perceptual and cognitive integration of cuts, they do not directly address the possibility that cuts function as articulatory units in a syntactic sense. These frameworks describe how continuity is achieved and how narrative cohesion is maintained, but they stop short of examining whether the cut itself may trigger recurrent neural mechanisms associated with segmentation or articulation. The approach of the cut as articulation contains the technical need for invisibility for the viewer and narrative integration as a necessity, both conditions consistent with previous studies from cognitive ecology. But it also aims to differentiate the most appropriate form of shot transition for the same event to be represented among all the possible ones that meet these two cognitive premises. Facing the same staging, countless possible shot combinations appear that will fulfill the premises defined by the AToCC and the SPECT; however, these combinations will produce different effects on the viewer.

To date, there are some experiments in this direction, analyzing how the shot change by cut affects the viewer depending on the shot scale (Cutting and Armstrong, 2016; Benini et al., 2019; Bálint et al., 2020). The use of different shot scales affects the mood conveyed to the viewers and their level of narrative engagement (Benini et al., 2019). Close-ups increase narrative engagement, character understanding, and emotional connection, while long shots decrease emotional engagement but improve narrative understanding by providing a broader spatial context. On the other hand, the transition between different types of shots in a cut has a greater impact on the viewer’s mood than maintaining the same type of shot during a cut.

Closer shots facilitate quicker identification of emotions, making them more suitable for episodes with high emotional intensity, optimizing the narrative according to the cognitive abilities of the viewers (Cutting and Armstrong, 2016). Consequently of this faster emotional discernment, close-ups tend to be shorter in duration than other shot scales where the face is smaller within the frame.

Close-up shots favor mental state attribution mechanics, which allow viewers to attribute mental states to the characters. However, this relationship is not linear, as the effectiveness of attribution does not increase consistently in the viewer as its frequency in the film increases (Bálint et al., 2020). The impact of this technical resource has a saturation limit point in the chaining of three close-up shots, resulting in its effect diminishing beyond this frequency. This condition implies that the excessive use of close-up shots can be counterproductive, even diluting their ability to generate an emotional connection between the spectator and the characters.

The different ways of designing the change of shot based on scale (Cutting and Armstrong, 2016; Benini et al., 2019; Bálint et al., 2020), as shown in previous studies, support the existence of an optimal articulation, which responds to structures inherent to the cinematographic medium and is defined through editing strategies and techniques. These studies demonstrate how the same event, depending on how the shots combination is articulated, can produce clearly differentiated effects on the spectator.

Building on this idea, the need to analyze how shots are structured within a cut invites a reconsideration of classical theories about the existence of a cinematographic language. Beyond the pursuit of invisibility in the cut and the seamless narrative integration of shots —both fundamental aspects of normative editing—it is essential to refine the understanding of how variations in other technical elements of film editing directly shape the spectator’s experience. These considerations, along with techniques to achieve invisibility and narrative coherence, have been extensively documented in editing manuals and essays, from foundational works (Eisenstein and Leyda, 1942; Bazin, 1958; Reisz and Millar, 1971; Dmytryk, 1984; Oudart, 2005/1969) to more contemporary works (Murch, 1995; Amiel, 2001; Marimón, 2015; Hullfish, 2017).

In this context, the present study aims to examine whether shot changes in continuity editing elicit neural responses that could support the hypothesis that editing functions as an articulatory mechanism. The goal is not to demonstrate that cinema constitutes a language in the strict sense, but rather to explore whether the cinematic cut fulfills articulatory functions associated with natural languages. Based on the theoretical proposition that editing connects different audiovisual units with meaning (Mitry, 1965), it is proposed that this function should be observable through recurrent neural dynamics, particularly in frequency bands associated with memory integration, temporal segmentation, and meaning construction (Klimesch, 1999; Ben-Yakov and Henson, 2018). Although the study does not claim that editing is equivalent to verbal language, it adopts an experimental hypothesis to test whether neural responses reflect a syntax-like articulation within the structure of the film.

### 1.1 Cinema as language

Practically since its inception, film studies has been exploring the nature of the shot change as an articulator, comparing it to the syntax of language (Shklovsky, 1971/1928; Eisenstein, 1977/1949). The earliest examples of this comparison can be found in assertions by filmmaker and film theorist Eisenstein, who stated that “montage is a syntax for the correct construction of each particle of a film fragment” (Eisenstein, 1977/1949, p. 111) or by film theorist, literary theorist and pioneer of Russian formalism Shklovsky, who argued that “montage is the syntax and etymology of the cinematic language” (Shklovsky, 1971/1928, p. 124). This idea of syntax responds to the organizing principle that gives meaning to moving images and relates them on a narrative, symbolic and emotional level. According to Eisenstein (1977/1949), cinematographic syntax is a dialectical process that links individual shots in a relationship that goes beyond simple narrative continuity, where the relationship between shots generates new meanings, operating as the equivalent of phrases and sentences in verbal language, but through editing. Shklovsky (1971/1928), for his part, argues that cinematographic syntax enhances the very form of cinema by foregrounding its technical elements—such as editing, framing, and rhythm—as carriers of meaning. In this way, cinema differs from the spectator’s usual perception of real events, modifying it in a process described as defamiliarization (*ostranenie*).

The theoretical proposal about the existence of an cinematographic language put forward by the Soviet formalists reached its peak development in the 1960s, when the film theorist and filmmaker Mitry and the pioneer of film semiotics Metz led a debate over how the cinematographic message is articulated. Both Mitry (1965) and Metz (1968) argued that cinema is a medium of expression that can convey emotions, and that it therefore has the capacity to create meaning for the receiver. Based on this capacity, both scholars asserted that film is semiotic in nature. Both Mitry and Metz stated that cinema responds to its own codes and conventions that can be systematically analyzed, defending the existence of a film grammar. However, it was on the nature of the medium of expression that the two theorists disagreed. While Mitry (1965) argued for the existence of a uniquely cinematographic language in which the cut represents the connection between units, Metz (1968), based on his ontological proposal of cinema dismissed the idea that the medium has a language of its own (defining it as a semiotic system), thereby rejecting the possibility proposed by Mitry (1965).

Metz (1968) adopts a semiological approach, influenced by structuralism and the theories of Ferdinand de Saussure and Roland Barthes. He is interested in cinema as a language, although he emphasizes that it cannot be considered a language in the strict sense. According to Metz, cinema is a system of signification based on codes that shares similarities with linguistic systems, but without being able to consider it a language. Metz classifies the different types of technical-narrative structures that appear in cinema, which he calls *grand syntagmas*. This classification attempts to describe how cinema organizes meaning, systematizing the narrative organization patterns that frequently appear in films. The syntagmas essentially reflect the relationships between shots through cinematographic editing and how these relationships contribute to the development of the narrative and the viewer’s perception of it.

Metz (1968), although identifies elements such as editing or camera movements as components of a film grammar, he describes the film medium as the outcome of the articulation of a set of devices that are relatively autonomous and absolutely essential. The direct consequence of this idea is that cinema cannot possibly have a signifying power of its own. Metz therefore denies the existence of minimal units of cinematographic meaning, and thus dismisses the possibility of a cinematographic language. The acceptance of this ontological hypothesis has epistemological consequences, as film analysis should be conducted by breaking the film up, not into inherently cinematographic elements such as shots, but according to the units that characterize the different independent signifiers that are articulated together.

For Mitry (1965), cinematographic language is unique because its meaning does not depend on an arbitrary relationship between signifier and signified, which makes cinema more intuitive and less dependent on abstract conventions. His interest lies in how cinema structures the spectator’s perception. Their analysis focuses on exploring the relationship between cinematic images and the viewer’s emotional or intellectual response. For Mitry, editing articulate the cinematic units of meaning and endowing them with significance while respecting the phenomenological experience of time. In this sense, editing acts as the main organizer of meaning in a film. It is through editing that images acquire meaning. The articulation between shots in editing refers to how different shots are organized, combined, and related in a cinematic sequence to construct a narrative, aesthetic, and emotional meaning.

Mitry (1965) positions his theoretical stance in opposition to Metz’s, as he considers that cinema in itself is an autonomous semiotic device and not an articulation of devices.^1^ He argues that all of the elements participating in the cinematographic device generate inseparable units without losing their ultimate meaning, and that therefore the signifier perceived by the spectator is unique to the cinematographic means of expression. In view of the existence of a unique signifier, Mitry (1965) concludes that a specifically cinematographic language does in fact exist. Therefore, by adopting Mitry’s perspective to conduct an analysis of a film, it can be divided into units of cinematographic language, such as the shot, taking all the parts that comprise it as a cohesive whole that generates a unique meaning, rather than as different independent signifiers that are joined together. The articulation of these minimal units of meaning through editing was what he defined as film grammar.

This debate has given rise to two completely irreconcilable positions in film theory that have continued to develop in parallel with each other with no prospect of a resolution. With the passage of time, beyond clarifying the debate, the position rejecting the existence of a cinematographic language has diversified to include stances such as those of Rivette (1951), Straub and Huillet (1984), Oubiña (2003) and Deleuze (1985), some of whom even dismiss the idea of cinema as the result of the articulation of different autonomous languages. On the other side of the argument, filmmakers such as Pasolini (1976), Egoyan (quoted in Tirard, 2005) and Wenders (2010), and theorists such as Bettetini (1963), Eco (1976), Martin (1955), Stam et al. (2005) and Bordwell et al. (1997) continue to support the theory that cinema is a language of its own, taking various perspectives that differ mainly in terms of their ontological definition of the smallest unit of cinematographic language.

According to Burch (1969), cinema is a system of signs that are articulated in a way comparable to a language. This articulation occurs through specific codes, such as editing, *mise-en-scène*, and sound. Burch’s proposal consists of defining cinema as a system of signs and cultural conventions that are learned and interpreted by the spectators. Therefore, the understanding of the film depends on a process of cultural learning, based on which viewers acquire the ability to interpret the meanings of cinematographic language according to their familiarity with the specific codes of the medium.

Burch (1969) proposed that, the smallest unit is the shot, which he defines as “pieces of space and time” based on which the narration is articulated. This is an idea that Bazin developed when he included the sequence shot in his theoretical framework, distinguishing the physical shot, which is a fragment of film between two cuts, from a more abstract conception of shot, to which he attributed the power to construct cinematographic language, and which he labeled the *image-fact*. Bazin identified the image-fact, defined as a relationship between terms in a specific period of time, to be the basic unit of a film. Mitry (1987) also considered shots that contain internal variations, defining a tracking shot as a series of units in a continuous filming sequence without cuts.

There are similarities to Bazin’s idea of the image-fact and Mitry’s series of units without cuts in the work of Arnheim (1932) and Kracauer (1960), who defined the frame as the smallest unit of meaning (frame as section of the shot with common conditions, different from frame as digital photogram). This would mean that a change of frame constitutes a transition to a new minimal unit. Bellour (1989) defines something similar when he suggests that the physical shot (the stretch of film contained between two cuts), which he refers to as a segment, may contain one or more *subsegments* in succession without the need of editing. The shot taken as a frame, image-fact or subsegment is defined by a formal variation that is not necessarily produced by an editing cut; instead, it is the product of a technical differentiation that gives rise to a meaningful change in the visual configuration of what is being depicted.

According to Bellour (2009), cinema articulates meanings through a set of visual, auditory, and narrative codes, integrated into a logic of representation and reading. This articulation occurs in the editing, as it allows for the organization of images and sounds in a sequence that constructs meaning. This organization follows certain cultural and structural rules, which makes cinema a system of signs comparable to a language. Bellour proposed that cinematic techniques such as cuts, camera movements, and transitions act as a grammar that regulates the viewer’s experience. In this model, the viewer not only perceives the filmed event but also interprets the signs of the film according to the implicit rules of the medium.

In addition to these strategies for dividing the shot in an effort to dissect a film into its smallest units of meaning is the theory proposed by Eco (1976) and Pasolini (1976), who identify the smallest unit of cinematographic meaning as what they call the *cinema*. According to Eco and Pasolini, the cinema, which constitutes the smallest, indivisible meaningful unit of audiovisual language, is defined by a variation in the meaning of the shot itself that does not necessarily coincide with a shot change or subsegment. Eco’s proposal is based on the fact that cinema is a semiotic medium that operates through a set of visual, auditory, and narrative signs that are articulated based on cultural codes and narrative conventions.

All of these ontological structures based on the acceptance of the idea of a cinematographic language, regardless of the specific theory adopted to determine what the smallest units of meaning are in the film medium, have the common feature of recognizing the shot change by cut as the site of a shift between contiguous units with different meanings. This means that the cut event is a physically identifiable transition that could be the subject of a neuroscientific experiment in a clearly defined timeframe to test the spectator’s perception of the cut.

### 1.2 Purpose and objectives

The study of cinema, since its origins, has analyzed the articulative nature of the shot change. The peak of this discussion occurred in the 1960s, when the debate arose over whether this quality allowed this articulation to be described as a language. This capacity attributed to the cut in terms of film construction ultimately refers to the very ontology of cinema, proving essential for its definition. This aspect was considered so clear that in the 1920s, cinematic movements emerged that regarded editing as the core characteristic that distinguished cinema from other artistic disciplines, giving rise to radical aesthetic movements such as abstract cinema (Léger and Murphy, 1924) or cinema pur (Chomette, 1926). These movements sought to articulate films purely through the editing of moving images, even going so far as to reject the very narration traditionally associated with the filmed event.

Therefore, it is considered that, in order to address the cinematographic ontology of film from the perspective of cognitive ecologism, it is necessary to address, in addition to those strategies that allow for the creation of a continuous virtuality (Smith, 2012) and a narrative integration of fragmented events (Loschky et al., 2020), an understanding of the articulation that occurs in the shot change by cut. Therefore, it is important to establish as a starting point the ongoing discussion about whether or not the existence of a cinematographic language can be considered.

Addressing the possible existence of a cinematographic language is a complex and overly ambitious endeavor for a single study. However, a manageable objective is to focus the analysis on the potential articulatory function of the shot change by cut in the photogrammatic construction. Therefore, the purpose of this study is to analyze the articulating role of the shot change by cut in cinematographic editing by identifying cognitive patterns in neural responses recorded through electroencephalograms (EEGs) of spectators while they are viewing such editing cuts. If the experiment identifies no neurological patterns associated with the articulating capacity of the cut, this would mean that these mechanisms should not be looked for in specifically cinematographic elements (such as, in this case, the cut) but in other independent signifiers that articulate the message. That is to say, while the presence of such neural response patterns would not resolve the debate about the existence of a cinematographic language, the absence of evidence defining the cut as an articulator would indeed support the stance that refutes its existence. Therefore, it is considered that the present study is a first step toward a possible series of studies that could outline a naturalistic-cognitive cinematographic ontology, taking as a theoretical starting point the classical perspective established by the debate on the possible existence of a cinematographic language.

To be able to define the shot change by cut as a cinematographic articulation, two conditions must be met. The first condition is that all the cuts must be perceived according to a common pattern of neural responses to the shot change, revealing specific mechanisms related to the comprehension and assimilation of the cut. The second condition is that each type of cut should produce specific variations in the neural patterns identified, in accordance with their particular taxonomic characteristics. It means that, once the neural reaction pattern triggered by the shot change event is identified based on all cuts collectively, it is expected that grouping the cuts by predefined typologies would reveal differences in the neural signals. These differences should specifically emerge in the regions of the neural response previously recognized as activated by the shot change event (based on all cuts collectively). The shot change by cut can only be considered an articulator of the film narration if the study identifies common patterns in the neural responses to the cut event and at the same time these patterns exhibit differences depending on the types of cuts. The study involves an EEG frequency domain analysis, focusing specifically on event-related desynchronization/synchronization (ERD/ERS). Frequency domain analysis has been shown to be effective in previous research for clarifying how the shot change by cut functions (Heimann et al., 2016).

This article represents the culmination of a series of publications that compile partial conclusions and address specific objectives of the research. The initial publications detail the analysis methodology employed, which differs from the conventional ERD/ERS analysis system (Sanz-Aznar et al., 2020a,b). The subsequent publication identifies common patterns in response to cuts through the study of ERD/ERS (Sanz-Aznar et al., 2021). Finally, the last publication examines the differential patterns associated with specific types of cuts using ERD/ERS analysis (Sanz-Aznar et al., 2023a). This last publication is complemented by an additional publication that reinforces the findings by analyzing neural patterns resulting from different cut types through an alternative methodology, specifically event-related potentials (2023b).

This article consolidates the specific objectives progressively addressed and integrates the entire process under the general objective of analyzing the cut as an articulating element from the perspective of a potential cinematic language. The study is based on the theoretical cinematographic framework that inspired the research and frames the discussion and conclusions within this context and perspective. This research developed is characterized by a markedly interdisciplinary approach, from a cognitive neuroscience perspective inspired by the debates of classical film theory on cinematic language. It does not aim to resolve the ontological question of whether cinema is a language in the linguistic sense, but rather to investigate whether editing cuts function as meaningful articulators of audiovisual structure, detectable through neural patterns. In this context, ERD/ERS analysis is used here to evaluate time-frequency brain dynamics in reaction to segmentation events. ERD/ERS analysis is used to evaluate time-frequency brain dynamics in reaction to segmentation events. Theta and delta band synchronization and desynchronization patterns have been linked to narrative segmentation, working memory engagement, and perceptual transitions (Silva et al., 2019), providing a suitable framework for testing whether different types of cuts elicit systematic and differentiated neural responses (Heimann et al., 2016; Andreu-Sánchez et al., 2018).

## 2 Materials and methods

The experimental process involved taking EEG recordings of film spectators while they were viewing film excerpts, and then processing and analyzing the frequency domain signal in order to identify neural response patterns common to the shot change by cut event, while at the same time detecting differences in these common patterns related to the specific type of cut.

### 2.1 Recording and processing the signal

The experiment provides continuous EEG recordings of 21 participating subjects (12 men and 9 women) while they were watching four different film excerpts. The subjects were between 22 and 38 years of age and reported no cognitive or psychological disorders. The mean age of the participants is 26.28 with a standard deviation of 3.74 and the mode is 27. The experiment was conducted at the Augmented Cognition Lab at Aalborg University, whereby the participants were mainly from this university background. The participants did not receive any financial reward for their participation, but were given a cinema ticket as a thank you.

The excerpts selected for this study were taken from the films Bonnie and Clyde (Penn, 1967), The Searchers (Ford, 1956), Whiplash (Chazelle, 2014), and On the Waterfront (Kazan, 1954). A post-experiment questionnaire asked participants which of the films they had previously seen in order to gauge their level of exposure to the film excerpts. Eleven of the 21 participants said they had never seen before any of the four films included in the study, while 10 said they had seen at least one. Bonnie and Clyde was the most frequently recognized title (seen by 9 participants), followed by Whiplash (6 participants), On the Waterfront (2 participants), and The Searchers (1 participant). Prior familiarity was relatively limited and dispersed, and no participant reported familiarity with all four clips. Additionally, no correlation was observed between familiarity and EEG patterns in a preliminary inspection, although future studies could explore this systematically.

**FIGURE 1:**
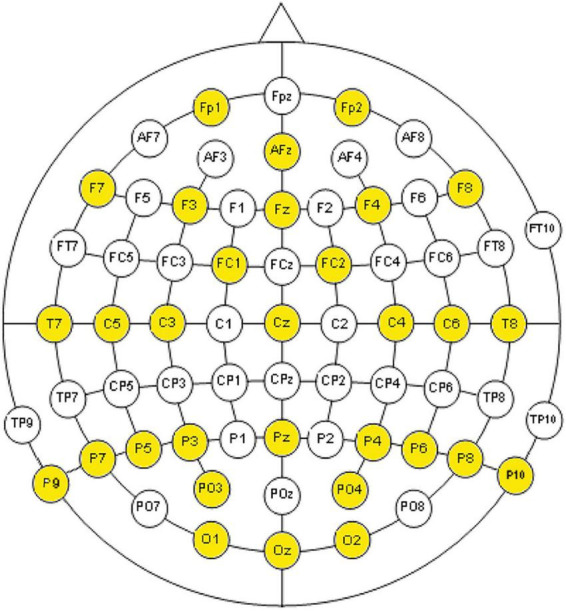
Placement of electrodes used according to the international 10-20 electrode placement system and the modified combinatorial nomenclature.

In order to record the electroencephalogram, 31 electrodes were placed as indicated in Figure 1. The amplifiers devices were two channel box g.Tec g.Gammabox (16 channels each one) connected to two biological amplifiers g.Tec g.USB Amp.

Since the experiments were conducted in the Augmented Cognition Lab at Aalborg University, the participants were drawn mainly from the university itself, with a predominance of international students fluent in English. Due to these conditions, the film footage chosen for the experiment was original footage in English, with no subtitles that might have affected perception (Perego et al., 2010).

For the study, film excerpt was taken from pre-existing films, considering it relevant, whenever possible, to analyze real films instead of clips prepared for the experiment. The film excerpts were chosen based on a series of selection criteria in addition to the language (more detail in Table 1). The excerpts selected belong to feature-length fiction films and all conform to the institutional mode of representation (IMR) defined by Burch (1991). At the same time, varied excerpts were sought, from different styles and aesthetics, as well as belonging to different structural moments of the film (first, second or third act, presentation of characters, conflict, etc.). Moreover, cinematographic language, if it does exist, is also shaped by the physical and technical possibility of adding a synchronous soundtrack to the film (Bordwell and Thompson, 1995). Since the aim of this study was to identify common neural patterns caused exclusively by editing cuts, it was more interesting to select excerpts from films that are markedly different in terms of techniques, pacing, and aesthetics (Sanz-Aznar et al., 2020a).

**TABLE 1:** Technical-aesthetic differences between film excerpts.

Film	Color or B&W	Rhythmic ratio^a^	Lighting^b^	Film style^c^
Bonnie and Clyde	Color	27.24	Modern	Transitional
The searchers	Color	4.79	Classical	Classical
Whiplash	Color	21.89	Modern	Post-classical
On the waterfront	Black and white	14.45	Classical	Classical

^a^Average cuts per minute in the excerpt.

^b^Lighting style (Revault and Allonnes, 1991/2003).

^c^Film style (Bordwell, 1985; Langford, 2009; Thanouli, 2009).

To facilitate spectator comprehension of the film excerpts, complete narrative units were chosen (McKee, 1997). It was decided to focus the study on continuity cuts, as these are the shot changes that are most likely to go unnoticed by the spectator (Smith, 2005; Drew and Soto-Faraco, 2024), which means that their detection and encoding patterns would be more immediate and unconscious. The shot changes by cuts selected therefore occur in the same spatial and temporal context. As the aim is to analyze the articulation between shots based on technical-aesthetical form and not narrative content, the study of cuts is focused on continuity editing, rejecting breaks in action, space and time. In total, the selected excerpts contained 261 shot changes by cut.

The neural recordings are compared based on taxonomic categories classifying the type of shot change by cut, for which two forms of taxonomic organization were established. Based on the shot scale and with respect to variation of the camera angle of the shot, a taxonomy was defined that only considers the shot immediately after the cut as a stimulus (G1), while a second taxonomy was designed that considers the variation between the shots immediately before and after the cut to define the stimulus (G2). Table 2 shows the different categories established for G1 and G2.

**TABLE 2:** Taxonomy for cuts grouped in G1 and in G2.

Taxonomy G1	Taxonomy G2
	Close-up to close-up
Medium shot with variation α ≥ 30	Medium shot to medium shot
Medium shot on axis	Wide shot to wide shot
Full shot with variation of α ≥ 30	Any to close-up
Close-up with variation of α ≥ 30	Any to wide shot
Close-up with variation of α < 30	Close-up or medium shot to full shot or wide shot
Wide shot with variation of α ≥ 30	Full shot or wide shot to close-up or medium shot
Wide shot on axis	Variation of α < 30
	Variation of α ≥ 30

G1 is focused on the post-cut input and G2 on the variations between shots.

**FIGURE 2:**
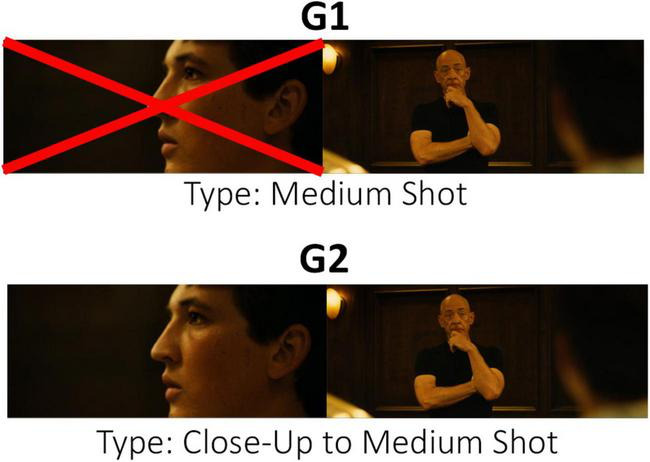
Example of taxonomic classification in G1 and G2 according to the shot change. Images taken from the *Whiplash* excerpt (Chazelle, 2014).

Figure 2 shows an example of the conceptual differences between taxonomies G1 and G2.

Once the neural signal had been recorded, after manually removing artifacts and analyzing ICA components, the signal was prepared for analysis. First, a model signal was generated based on the 21 subjects of analysis per electrode for each film excerpt. Once this signal had been averaged, the study focused on the area of interest, which was 1 s before and 1 s after each cut, resulting in 2-s time windows. These time sections were used to create the ASF (average signal per film) and ASC (average signal per cut) model signals. ASF model signals are oriented toward detecting common patterns of neural responses to the cut event, while ASC model signals are designed to identify variations in these neural patterns based on the specific type of shot change.

ASF model signals are comprised based on all the shot changes by cuts included in the recorded signal for each film excerpt. In this way, four ASF model signals per electrode are obtained for each excerpt. Each ASF has a duration of 2 s, with the shot change in the central instant.

ASC model signals are comprised based on all the shot changes by cuts in the same film excerpt that can be classified as the same type of shot change. Thus, for each electrode and specific type of shot change, four ASC model signals were obtained, one for each film. As two taxonomies (G1 and G2) were established, ASC signals were generated for both G1 and for G2. Like the ASFs, each ASC has a duration of 2 s, with the cut in the center.

Although G2 involves a more complex processing load than G1, since it includes both the shot preceding and following the cut, it is precisely this condition that allows for the evaluation of whether the cognitive system processes the cut relationally, rather than merely as an isolated perceptual event, as modeled in G1. Including G2 allows to analyze if the brain encodes the cuts as a meaningful change, activating working memory or predictive mechanisms. Previous studies on event segmentation and narrative updating (Ben-Yakov and Henson, 2018), are coherent with the notion that editing cuts function as articulatory boundaries within film sequences.

Once all the ASF and ASC model signals had been generated, they were transformed from the frequency domain by means of a fast Fourier transform and broken down according to the frequency ranges to be analyzed. The frequency bands they were broken down into were: delta (0.5–3 Hz), theta (3–7 Hz), alpha (7–14 Hz), beta (14–32 Hz) and gamma (32–42 Hz). These frequency bands were then subdivided into the following ranges: low delta (0.5–1.5 Hz), high delta (1.5–3 Hz), low theta (3–5 Hz), high theta (5–7 Hz), low alpha (7–10.5 Hz), high alpha (10.5–14 Hz), low beta (14–23 Hz), high beta (23–32 Hz), low gamma (32–37 Hz) and high gamma (37–42 Hz). In total, the ASF and ASC model signals for each electrode were broken down into 15 defined frequency ranges (5X3—full, high and low per each frequency band).

### 2.2 Statistical analysis

The statistical analysis consisted of two stages. The first involved the definition of the patterns of common neural responses to the shot change by cut using the ASF model signals. This was followed by an analysis using the ASC model signals to identify variations occurring within these patterns based on the specific type of cut.

To identify common neural patterns of responses to the cut event, pairwise comparisons were made of the same electrode in the same frequency band between model signals from different film excerpts. Based on four ASF model signals, a total of six comparisons were made. These pairwise comparisons were analyzed for correlation and dependence using a Spearman correlation test (Rho > 0.5) and an exact permutation test (*p* < 0.05). The combination of Spearman tests and exact permutations test increases the robustness and precision of the results, minimizing type I errors (false positives), in addition to ensuring that the correlations detected are statistically significant. Furthermore, to increase the demand for robustness in the results minimizing type I errors, a neural response pattern triggered by the cut was assumed only if significant positive correlation and dependence were found in all six possible comparisons.

The comparisons were made using sliding time windows, comparing the same time window of the same electrode in the same frequency band among the four ASFs. Each time window is comprised of six consecutive samples. The central window, marked as 0 ms, is comprised of three samples before and three samples after the cut event. Given that the EEGs were recorded at 256 Hz, the Fourier transform took ranges of eight EEG samples, and each time window took six frequency domain samples, each time window represents an interval of 187.5 ms. This process used to identify neural response patterns can be consulted in previous publications where it is explained in more detail (Sanz-Aznar et al., 2020b; Sanz-Aznar et al., 2021).

After completing the time windows in the electrodes and frequency bands where neural response patterns triggered by cuts were identified, the variations occurring between different types of cuts were analyzed for the G1 and G2 taxonomies. Based on these, the ASC model signals were generated. To identify the variations, a Kruskal-Wallis analysis of variance (significance threshold: *p* < 0.05) was performed together with a Tukey-Kramer multiple comparison test (significance threshold: *p* < 0.05), taking the type of shot change as a comparison condition (see Table 2). Both methods were applied using sliding windows of six samples for each electrode and frequency range.

The Kruskal-Wallis analysis of variance between different ASCs made it possible to identify time windows, electrodes and frequency bands where differences occur in at least one specific type of cut despite the existence of correlation and dependence in the ASF comparisons. In order to identify the types of cut between which this variation is occurring, a *post hoc* Tukey-Kramer multiple comparison test was performed. The application of this *post hoc* analysis also allowed us to eliminate potential errors in the analysis of variance. This process used to identify variations in neural response patterns based on the cut category can be consulted in previous publications where it is explained in more detail (Sanz-Aznar et al., 2023a).

The analysis was carry on without pre-selecting regions of interest. The electrodes reported as relevant were not chosen a priori but emerged from a full-brain exploratory analysis as those consistently showing significant effects across multiple conditions and comparisons. The highlighted electrodes were due to their involvement in statistically significant neural responses as defined by the stringent criteria of correlation and dependence across comparisons.

Once the relevant time windows were identified for each electrode and frequency range, the results were analyzed in terms of event-related desynchronization/synchronization (ERD/ERS). ERD/ERS involves comparing the power (energy) in a given frequency band during a specific condition (after the cut) against a baseline period. In the case of this study, the second prior to the cut was established as the baseline, as the aim in analyzing the articulation between two shots joined by a cut was to study the variation caused by the shot change in the temporal continuum of the film and not to a neutral state of rest unrelated to the film. To obtain the ERD/ERS value, Equation 1 (Klimesch, 1996; Doppelmayr et al., 1998) was used:


$$E\,R\,D=\frac{B\,a\,s\,e\,l\,i\,n\,e-T\,e\,s\,t}{B\,a\,s\,e\,l\,i\,n\,e}\,100$$

In Equation 1, *Baseline* is the average power of the frequency band of interest during the baseline interval and *Test* is the average power in that same frequency band during the post-cut interval being examined. The result of the equation is expressed as a percentage, where desynchronization (ERD) processes corresponded to positive values resulting from this operation and synchronization (ERS) processes corresponded to negative values. ERD typically reflects increased cortical activation at a particular frequency when actively processing new information, and ERS indicates a reorganized neuronal firing pattern in that frequency band, often linked to less active processing or a reset mechanism.

## 3 Analysis of results

**FIGURE 3:**
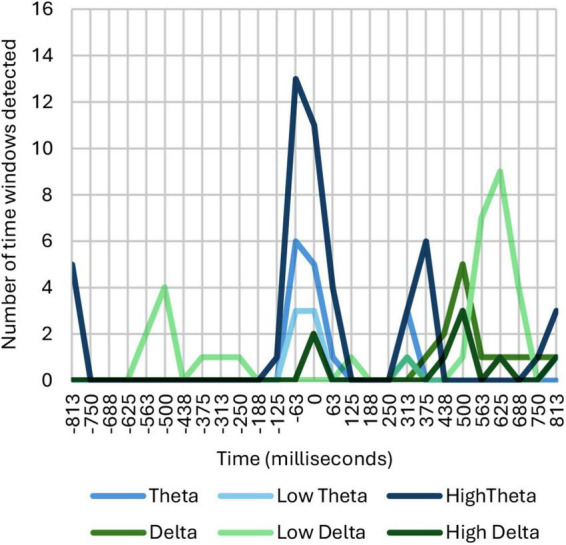
Number of time windows showing correlation and dependence among ASFs, broken down into theta and delta ranges.

The initial results were obtained by adding up all those windows on the timeline where identified significant correlation and dependence, as shown in Figure 3.

As can be seen in Figure 3, common neural responses appear in the theta and delta frequency bands in different time intervals. An ERD/ERS analysis reveals a synchronization in theta between 0 and 200 ms, a desynchronization in theta between 200 and 500 ms, a desynchronization in delta between 500 and 700 ms, and a synchronization in theta between 650 and 1,000 ms. The activity detected in delta is located in the middle region of the parietal, frontal, frontocentral and central areas with no signs of lateralization. On the other hand, the activity detected in theta is located mainly in the parietal area, although in the case of high theta it also extends through the central, occipital and middle regions of the frontal and frontocentral lobe. Moreover, in the synchronization occurring in theta during the first 200 ms, a degree of left lateralization was detected.

Once the neural response patterns were identified, an analysis of variance was performed to detect differences occurring based on the specific type of cut. This analysis was conducted on the G1 taxonomies, based on the new shot as the absolute stimulus (ignoring the previous shot), and on G2, based on analyzing the variation between the shots immediately before and after the cut. In the analysis of variance of those time windows where significant correlation and dependence occurs, a random comparison between different groupings of shots should not give positive results, as variances should only be identified if the different cuts comprising each taxon exhibit behavior that is homogeneous within that taxon but different from those comprising the other taxa.

**FIGURE 4:**
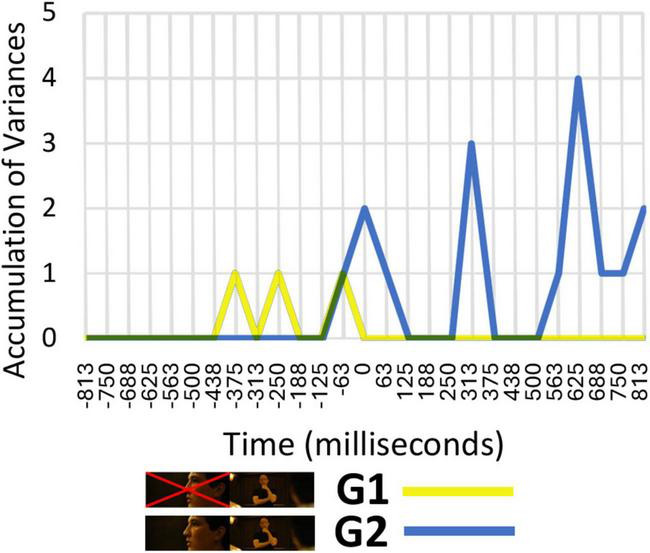
Number of time windows for the same electrode and frequency range showing significant differences between different types of cuts.

As can be seen in Figure 4, variations in neural patterns based on the specific type of shot change cannot be identified in the G1 taxonomy, as the responses are the same as they would be if the taxonomy were constructed using random groupings of cuts. However, differences between different types of cuts can be identified in the G2 taxonomy, which means that this taxonomy can be considered to be in keeping with the way the cognitive system processes shot changes, and not to constitute a random grouping of cuts at the neural level. These findings suggest that, at a cognitive level, the neural system processes the shot change information in a relational manner. Rather than simply replacing the outgoing information with the incoming one, it actively compares the difference between shots. If the positive results had accumulated for G1, that would have indicated the opposite conclusion, namely, that only the incoming shot needs to be processed, without taking the outgoing shot into account.

The time windows in which variations were identified are associated with theta and high theta frequency ranges between 0 and 400 ms and with delta and low delta between 500 and 1,000 ms. In the period between 0 and 400 ms, variances were detected in the central and posterior regions (Cz and Oz), while in the period between 500 and 1,000 ms variances were detected in the more frontal (F3, Afz, F8) and parietal regions (P9 and P7), exhibiting left lateralization.

By analyzing the types of cuts that produce variances between them using the Tukey-Kramer multiple comparison test, it is possible to detect results that can be grouped according to the nature of the variation between shots. Table 3 shows the new reorganization based on the results obtained from the Tukey-Kramer multiple comparison test.

**TABLE 3:** Electrodes that are differentiated with taxonomies grouped by shot scale variation.

	Same scale	Increased scale	Reduced scale
**0 – 125 ms**			
Same scale	Cz(2)/OZ(1)		
Increased scale	Oz(1)/Cz(2)	P3(1)	
Reduced scale	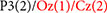	P3(1)	∅
**250 – 375 ms**			
Same scale	F8(1)		
Increased scale	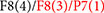	∅	
Reduced scale	P7(2)/F8(2)	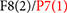	P7(2)
**500–1,000 ms**			
Same scale	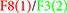		
Increased scale	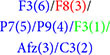	∅	
Reduced scale	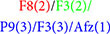	P7(1)/C3(2)	∅

Electrodes detected in the **theta** frequency band appear in black, those detected in the 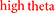 in red, those detected in the 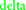 in green, and those detected in the 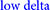 in blue. The number of times each electrode showed significant differences is indicated in parentheses.

Groupings are made on the basis of three groups, which are; same scale (including close-up to close-up, medium shot to medium shot and wide shot to wide shot), increased scale (including any shot to wide shot and close-up or medium shot to full shot or wide shot) and reduced scale (including any shot to close-up and full shot or wide shot to close-up or medium shot).

The greater number of differences appear between categories of different groups, while comparing the categories within each group produces fewer differences between them. The time range that is most relevant in this comparison is between 500 and 1,000 ms.

## 4 Discussion

The identification of common patterns of responses to the shot change event would not be enough on its own to define the cut as an articulator, but it is sufficient to define it as a detectable cognitive event. To be able to define the shot change by cut as an articulation, in addition to constituting a cognitive event, it would have to be possible to differentiate it based on an established taxonomy. The analysis of the results related to the identification of neural patterns common to all types of cuts indicates that the condition necessary to attribute an articulating function to the cut has been met. At the same time, it has also been possible to confirm that this common response pattern displays a variation in the neural response depending on the specific type of cut. As both these conditions have been confirmed in this study, it’s possible to define the cut as a cinematographic articulator.

The neural response pattern producing the widest variation in neural rhythms was detected in the delta frequency ranges between 375 and 750 ms, especially in low delta. The desynchronization identified in delta rhythms is believed to be related to cognitive processes, reflecting the suppression of the default mode network (Dimitriadis et al., 2010). Deactivation of the default mode network (DMN) is associated with the beginning of a conscious activity, which could be related to the theory posited by Mitry (1965) about the demands that the shot change places on the spectator. For Mitry, the shot change elicits intellectual activity in the spectator, while the shot itself engages the spectator emotionally. In this sense, Li et al. (2014) associate DMN with emotional perception and empathy in social relations.

The neural response pattern identified as occurring most immediately after the cut event is in the theta frequency band, especially in high theta. This dependence detected in the theta range corresponds to a synchronization in the first 188 ms after the cut. The parietal region was identified as the main region responsible for the detection of the cut, with variations in its frequency over time, a phenomenon associated with visual-spatial memory (Wolinski et al., 2018). In this first 188 ms, a tendency toward left lateralization was identified in neural responses, something that is typically associated with cognitive language processes (Redolar, 2014). Theta frequency band synchronization during the first 250 ms is also associated with unconscious emotional processing of stimuli (Knyazev and Slobodskoj-Plusnin, 2009).

On the other hand, synchronization in the theta band is considered mainly to be a reflection of activity in the hippocampus and is associated with encoding of new information and memory-related processes (Klimesch, 1999; O’Keefe and Recce, 1999; Shirvalkar et al., 2010). Activity in the frontal region, both for memorizing and for remembering, is reflected in theta frequencies (Jensen and Tesche, 2002). In this sense, the results point to a neural response pattern consistent with hippocampal activity and align with a previous MRI study showing that shot changes trigger activity in the hippocampus (Ben-Yakov and Henson, 2018).

Various studies locate time perception in the hippocampus and associate this part of the brain with memory processes (Howard and Eichenbaum, 2015; Eichenbaum, 2017) through so-called “time cells” (MacDonald et al., 2011). Along these lines, it has been suggested that the hippocampus is responsible for encoding time through memory (Teki et al., 2017). Based on the results of this study, these conclusions support the hypothesis that filmic time is constructed by means of the cut (Deleuze, 1983), reinforcing the idea that editing is responsible for the articulation of narrative discourse in film. Films have a specific physical duration that does not usually correspond to the length of filmic time, which constructs a time of its own in the narration. Filmic time is a perception that has to be encoded (Deleuze, 1983; Deleuze, 1985), just as Teki et al. (2017) propose that our perception of the reality that surrounds us is encoded in memory, making the hippocampus responsible for encoding reality in a temporal structure.

Some studies also associate the hippocampus with the encoding of space in a manner similar to time encoding (Staresina and Davachi, 2009; Olton et al., 1979; Eichenbaum, 2014). According to these theories, the hippocampus fulfills the function of space-time encoder, an idea in keeping with Burch’s (1969) description of the need to decode films. Burch defines the film as an abstraction of two *découpages*: temporal *découpage* and spatial *découpage*. For Burch, a film is a succession of shots, where each shot is a fragment of space and time, and the filmic discourse is articulated by encoding these spatial and temporal fragments to create the virtual domain of filmic space and time. The results suggesting that the cut triggers synchronizations in theta could be related to the memory processes of the hippocampus, which means that time and space perception may be encoded through the cut.

The finding of left lateralization in theta neural activity to process editing cuts is worth considering in relation to studies of how the brain processes language. Specifically, the fact that left lateralization toward the parietal region is typically associated with cognitive language processes (Redolar, 2014) may point to a connection between filmic perception and linguistic perception. This possibility returns us once again to the debate begun in the 1960s by Mitry (1965) and Metz (1968) about the possible existence of a cinematographic language. Since the earliest days of film theory, various theorists and filmmakers have been developing possible explanations of what cinema is based either on the existence of a cinematographic language (Bettetini, 1963; Pasolini, 1976; Stam et al., 2005; Bordwell et al., 1997; Wenders, 2010) or on the impossibility of its existence (Rivette, 1951; Straub and Huillet, 1984; Deleuze, 1985; Oubiña, 2003). Although the purpose of this study has not been specifically to compare cognitive processes of cinematographic and linguistic perception, the results do suggest that it may be worthwhile to explore potential connections with neuroscientific studies focusing on language processing (Spironelli and Angrilli, 2010; Morillon et al., 2010; Titiz et al., 2017).

Of the two taxonomies initially proposed for study, the G1 group is exclusively dependent on the first frame after the cut, while the G2 group is a categorization based on the relationship between the frames located around the cut. An analysis of the differences between the categories established reveals that the cuts grouped according to the G2 taxonomy display cumulative results in periods of concentration over the course of time after the cut event, while in the cuts grouped according to G1 the results exhibit no differences from a random grouping of cuts. This suggests that the assimilation of the cut by the spectator does not depend exclusively on the new shot, but instead is associated with the relationship between the shots before and after the cut. These results are coherent with other research that defines that differences in the intensity of the effect on the viewer’s mood if the cut changes the scale or if it keeps the same type of scale (Benini et al., 2019).

The results of the comparison of G1 and G2 clearly tie in with the theories of shot changes as cinematographic articulators between two units (Mitry, 1965; Burch, 1969). The dependence between shots results compatible with the argument that film is a form of language, in which meaning is produced by the combination of signifiers (Mitry, 1965; Burch, 1969; Bellour, 1989). Mitry (1965) defines film as having a fleeting symbolic nature, where there is no stable relationship between signifier and signified, as the signified is the result of the signifier and its context. As a result, the convention of the symbol is replaced in cinema with fleeting symbolic values whereby ideas can be signified in multiple ways, and at the same time they cannot always be signified in the same way. The shot is therefore a unit of space and time that is resignified in its combination with another shot by means of the cut.

This way that the brain has of processing information is consistent with that described in feature integration theory, where each stimulus is compared with the previous perception (Treisman and Gelade, 1980). It is also in line with the Boolean map theory of perception, which is based on the processing of the variation perceived in the image processed previously (Huang et al., 2007), as well as with theories of discrete perception (Freeman, 2006; VanRullen et al., 2007; Busch and VanRullen, 2010) based on a discontinuous attentional system that creates a sensation of continuity between the instants perceived (Koch, 2004). This sensation of an integrated reality is the product of memory retrieval processes (Huang et al., 2007).

The analysis of the results revealed that neuronal responses varied depending on changes in scale value during shot transitions. These findings were consistent with those obtained from the same dataset using an event-related potential (ERP) analysis in a separate study (Sanz-Aznar et al., 2023b). By analyzing the neuronal signals in the time domain, which allowed for the study of ERPs, distinct reaction patterns were observed between groups with the same scale, increased scale, and reduced scale cuts, similar to the results obtained through ERD/ERS analysis. This differentiation, based on the variation or continuity of shot scale, was a common consideration in film editing manuals, which aimed to manage the narrative flow according to the emotional impact intended to be conveyed to the viewer (Reisz and Millar, 1971; Marimón, 2015).

The present study proposes an approach toward considering the cut as a cinematic articulator, aiming to recover from an experimental perspective the debate on the possible existence of a cinematographic language. As such, clear limitations appear in the study that should be addressed with complementary research that broadens the spectrum of possibilities allowed by film editing and refines the generated knowledge. The present research has been applied solely with cuts in continuity, maintaining spatial and temporal unity. This implies that the results are limited to this sample universe until they are verified on different types of cuts not included in the present study. Moreover, the present study focuses on basic articulation, without applying a comparison of neural patterns related to specific linguistic processes with the obtained neural responses. However, the results of the present study open the door to this type of comparisons from an experimental justification, complementary to the already existing cinematographic theories that advocate for the existence of a cinematographic language.

## 5 Conclusion

The shot change by cut event can be identified at the neurological level thanks to response patterns in the theta and delta frequency bands. The method described above can yield results specifically associated with the perception of the shot change, supported by previous research that also identifies activity in the hippocampus (Ben-Yakov and Henson, 2018). The neural rhythm modulation detected in delta, associated with the default mode network (Dimitriadis et al., 2010), along with the modulation detected in theta, associated with encoding processes and accessing memory (Klimesch, 1999), suggests that to assimilate the shot change the spectator’s cognitive system may require a neural process associated with encoding and decoding, especially given the detection of balanced theta modulations in the left hemisphere of the brain associated with language processing (Bzdok et al., 2016). The relationship between the results of this study and hippocampus activity is considered to be of special interest because of the neurological theories that link the hippocampus to our spatial-temporal encoding of the reality that surrounds us.

As the structure of the G2 taxonomy’s categories is based on the differences between the shots immediately before and after the cut and this grouping gives rise to different neurological responses observable between categories, it’s possible to infer an absolute dependence of the spectator’s neurological response on the differences identifiable between the two shots. This situation suggests that the spectator’s comprehension of the cut may be dependent on the shots involved in the shot change, and not solely on the specific input of the new shot that follows the cut (G1). Specifically, differences were identified in neural processes depending on variations between shot scales before and after the cut. This supports classical theories proposing the existence of a cinematographic language, although further experiments are needed to be able to draw any conclusions on the question. Based on the current state of research, the shot change can be described as an articulation of signifiers (Mitry, 1965) in an integrated reality (Huang et al., 2007) based on a discontinuous system (Koch, 2004).

Regarding possible future research, there is the possibility of conducting experiments more oriented toward locating neural patterns correlatable with purely linguistic processes; however, it is considered that before taking this step, two verifications need to be carried out: The first involves replicating the experiment on cuts that do not respect spatial and temporal unity. The second is to replicate the experiment by looking for points of articulation not in the shot change by cut, but in variations that can be considered changes of unit within the shots, as for example Bellour (1989) proposes with the concept of subsegments. Only after these two verifications does it truly make sense to delve deeper into the search for patterns correlatable with linguistic processes and to propose experiments that would ontologically define what the minimum cinematic unit with its own meaning would be.

## Data Availability

Publicly available datasets were analyzed in this study. This data can be found here: https://osf.io/exbt7/?view_only=a0427e4b8cee4c51bb09b0f978b95883.

## Footnotes

1 It is a semiotic device in the sense that it is a formal structure that allows us to perceive and transform information, similar to Jackendoff (1987) *conceptual structure*. Baudry (1975) describes the cinematographic device as all those aspects of a film that regulate the relationship between the spectator and the work to create an impression of reality.
